# In Vivo Safety and Efficacy of Thiosemicarbazones in Experimental Mice Infected with *Toxoplasma gondii* Oocysts

**DOI:** 10.3390/biomedicines13081879

**Published:** 2025-08-01

**Authors:** Manuela Semeraro, Ghalia Boubaker, Mirco Scaccaglia, Dennis Imhof, Maria Cristina Ferreira de Sousa, Kai Pascal Alexander Hänggeli, Anitha Löwe, Marco Genchi, Laura Helen Kramer, Alice Vismarra, Giorgio Pelosi, Franco Bisceglie, Luis Miguel Ortega-Mora, Joachim Müller, Andrew Hemphill

**Affiliations:** 1 Department of Veterinary Science, University of Parma, Via del Taglio, 10, 43126 Parma, Italy; marco.genchi@unipr.it (M.G.); alice.vismarra@unipr.it (A.V.); 2 Institute of Parasitology, Vetsuisse Faculty, University of Bern, Länggassstrasse 122, 3012 Bern, Switzerland; boubaker.gha@gmail.com (G.B.); maria.ferreira@unibe.ch (M.C.F.d.S.); kai.haenggeli@unibe.ch (K.P.A.H.); joachim.mueller@unibe.ch (J.M.); andrew.hemphill@unibe.ch (A.H.); 3 Department of Chemistry, Life Sciences and Environmental Sustainability, University of Parma, Parco Area Delle Scienze, 11/a, 43124 Parma, Italy; mirco.scaccaglia@unipr.it (M.S.); giorgio.pelosi@unipr.it (G.P.); franco.bisceglie@unipr.it (F.B.); 4 SALUVET, Animal Health Department, Faculty of Veterinary Sciences, Complutense University of Madrid, Ciudad Universitaria s/n, 28040 Madrid, Spain; luisucm@ucm.es

**Keywords:** *T. gondii*, gold-based compounds, thiosemicarbazones, in vivo

## Abstract

**Background**: *Toxoplasma gondii* is a globally widespread parasite responsible for toxoplasmosis, a zoonotic disease with significant impact on both human and animal health. The current lack of safe and effective treatments underscores the need for new drugs. Earlier, thiosemicarbazones (TSCs) and their metal complexes have shown promising activities against *T. gondii*. This study evaluated a gold (III) complex C3 and its TSC ligand C4 for safety in host immune cells and zebrafish embryos, followed by efficacy assessment in a murine model for chronic toxoplasmosis. **Methods**: The effects on viability and proliferation of murine splenocytes were determined using Alamar Blue assay and BrdU ELISA, and potential effects of the drugs on zebrafish (*Danio rerio*) embryos were detected through daily light microscopical inspection within the first 96 h of embryo development. The parasite burden in treated versus non-treated mice was measured by quantitative real-time PCR in the brain, eyes and the heart. **Results**: Neither compound showed immunosuppressive effects on the host immune cells but displayed dose-dependent toxicity on early zebrafish embryo development, suggesting that these compounds should not be applied in pregnant animals. In the murine model of chronic toxoplasmosis, C4 treatment significantly reduced the parasite load in the heart but not in the brain or eyes, while C3 did not have any impact on the parasite load. **Conclusions**: These results highlight the potential of C4 for further exploration but also the limitations of current approaches in effectively reducing parasite burden in vivo.

## 1. Introduction

*Toxoplasma gondii* (*T. gondii*) is a globally widespread parasite responsible for toxoplasmosis, a zoonotic disease that significantly affects both human and animal health [1]. Its life cycle includes three different stages: (i) sporozoites, encapsulated in oocysts that are formed within their definitive hosts (felines) and are shed through the feces; (ii) tachyzoites, representing the rapid proliferative stage that causes acute disease; (iii) bradyzoites, the slowly proliferative stage, forming tissue cysts and causing chronic disease [2,3]. In humans, the infection can lead to severe complications, particularly in immunocompromised individuals [4] and pregnant women, where it may cause life-threatening illnesses or congenital abnormalities [2,5]. Postnatal transmission of *T. gondii* to humans is mainly through ingestion of either tissue cysts in raw or undercooked meat or oocysts that contaminate food, water and soil [6,7]. Contamination of water by oocysts of atypical strains of *T. gondii*, for example, has been reported as responsible for severe outbreaks of acute toxoplasmosis in Brazil [8,9]. In animals, especially livestock, *T. gondii* infection can result in economic losses due to abortions and stillbirths mainly in sheep [10,11]. The parasite’s ability to infect a wide range of warm-blooded hosts and establish persistent infections contributes to its extensive prevalence and impact worldwide [6]. The current drugs used against toxoplasmosis have several limitations [12]. Combined pyrimethamine–sulfadiazine plus leucovorin is the first-line treatment but is prone to causing adverse side effects in many patients. Other treatment options include pyrimethamine–clindamycin or trimethoprim–sulfamethoxazole, and pyrimethamine or sulfadiazine can also be combined with atovaquone or azithromycin [13,14,15]. Severe adverse reactions, some resulting in fatal outcomes, have been described [16]. Thus, safer and more-efficacious drugs are needed. Thiosemicarbazones (TSCs) are organosulfur compounds known for their antimicrobial and anticancer properties [17,18,19,20,21,22,23]. Two specific TSC iron chelators, Dp44mT and DpC, belonging to the di-2-pyridylketone TSC (DpT) class, demonstrated significant activities against cancer cells both in vitro and in vivo. These compounds are believed to accumulate in lysosomes, leading to the generation of reactive oxygen species (ROS) and inducing apoptosis [24]. The biological properties of semicarbazones and TSC–metal conjugates are often related to metal ion coordination, with the metal complex being more active than the free ligand [25]. Side effects may decrease upon complexation, or the complexes may exhibit bioactivities that are not shown by the free ligand [17]. Several TSC derivatives have been tested for activity against *T. gondii* in vitro, with several showing promising results against the proliferative tachyzoite stage [26,27,28,29,30,31,32,33]. Gold-based complexes have attracted significant interest for treating *T. gondii* infections. A notable example is auranofin, an FDA-approved drug for rheumatoid arthritis [34], which has been repurposed as an antibacterial agent [35] and was shown to exhibit high efficacy against *T. gondii*, both in vitro and in an experimental model of acute toxoplasmosis [36]. A more recent study demonstrated potent activities of 4-chlorophenylthioacetone-derived TSCs against *T. gondii*, leading to the increased survival of mice experimentally infected with *T. gondii* [37]. We recently investigated the properties of three gold(III) complexes (C1, C2 and C3) and the respective salicyl-TSC ligand C4 with respect to in vitro activity against *T. gondii* tachyzoites. Upon short-term exposure (3-day assays), C3 and C4 were shown to exhibit promising IC_50_ values in the lower nanomolar range (<30 nM), and Transmission Electron Microscopy (TEM) detected transient structural alterations in the parasitophorous vacuole membrane and the tachyzoite cytoplasm. However, treatments carried out for extended periods of time showed that these drugs could initially attenuate proliferation of *T. gondii* tachyzoites but did not act parasiticidal in vitro [38]. In this study we set out to investigate the safety and potential applicability of these two compounds in an in vivo experimental mouse model.

## 2. Materials and Methods

### 2.1. Cell Culture Media, Biochemicals, Thiosemicarbazones

Culture media were purchased from Gibco-BRL (Zürich, Switzerland) and biochemicals from Sigma (St. Louis, MO, USA). Thiosemicarbazones (TSCs) were originally synthesized at the Department of Chemistry, Life Sciences and Environmental Sustainability, University of Parma, Italy [38]. For in vitro studies, stock solutions of 20 mM were prepared in dimethyl-sulfoxide (DMSO) and stored at −20 °C.

### 2.2. Host Cells and Parasites

Human foreskin fibroblasts (HFFs; PCS-201-010™) were maintained as described [39]. *T. gondii* oocysts of the type II isolate TgShSp1 [40] were obtained from Complutense University of Madrid, Spain, and stored at 4 °C until use.

### 2.3. Assessment of Viability of Murine Splenocytes After Treatment with C3 and C4 In Vitro

Spleens from naîve or *T. gondii*-infected mice were aseptically collected from mice after euthanasia. Splenocytes were isolated as previously described [39] and seeded in 96-well plates (5 × 10^4^ cells/well). The splenocytes were either left unstimulated (negative control) or stimulated with Concanavalin A (ConA) (5 mg/mL) or Lipopolysaccharides (LPS) (10 mg/mL), which are known to activate T and B cells, respectively. The compounds being tested were added to the cultures at concentrations of 0.1 µM, 0.5 µM, 1 µM and 2 µM. Cyclosporin A (CsA) was included as a positive control for strong immunosuppressive activity. After 48 h of culture at 37 °C/5% CO_2_, the splenocytes’ viability was assessed using AlamarBlue assay as described before [39]. Statistical analyses were performed using Microsoft Excel® software package (Microsoft, Redmond, WA, USA).

### 2.4. Assessment of C3 and C4 Interference in Early Zebrafish Embryo Development

A zebrafish (*Danio rerio*) embryo development assay was carried out following the methodology described by Anghel et al. [41] and Boubaker et al. [39]. The tested concentrations were 20 µM, 10 µM, 1 µM and 0.2 µM for both compounds. In the negative control plate, embryos were placed in osmosis water, while in the solvent control plate, embryos were placed in osmosis water containing 0.1% DMSO. The embryos were observed, and malformations or viability changes were recorded at 24, 48, 72 and 96 hpf. At the end of the experiment, the survival index (Si) was calculated for each treatment condition as described by Anghel et al. [41] and Boubaker et al. [39]. The Si is calculated by subtracting Smean from Sassay. A negative Si indicates interference, while Si ≥ 0 implies no interference in embryo development.

### 2.5. C3 and C4 Treatments of CD1 Mice Orally Infected with TgShSp1 Oocysts

Prior to the experiment, all the mice were randomly assigned to different treatment groups: Solvent control group (Solvent ctr): infected and treated with corn oil/2.5% DMSO (*n* = 8); C3 treatment group (C3): infected and treated with C3 at 10 mg/kg/day emulsified in corn oil/2.5%DMSO (*n* = 8); C4 treatment group (C4): infected and treated with C4 at 10 mg/kg/day emulsified in corn oil/2.5% DMSO (*n* = 8). Peroral infection with 120 TgShSp1 oocysts suspended in 100 µL PBS was achieved by gavage at day 0 [39]. Treatments by oral gavage with corn oil/2.5% DMSO or drugs emulsified in corn oil/2.5%DMSO (100 µL/treatment) were initiated on day 3 postinfection (p.i.) and repeated once daily for 4 consecutive days, 5 days overall. During the entire experiment, mice were monitored for potential health issues at least twice per day. All the mice were sacrificed at 30 days p.i. Brain, eyes and heart were collected and stored at −20 °C for subsequent DNA extraction and quantification of *T. gondii* load by quantitative PCR (qPCR). The spleen was collected, and splenocytes were isolated and cultured as described below.

To study the potential impact of C3 and C4 treatments on splenocytes of naîve mice, two non-infected groups of 8 mice each were treated with C3 and C4 as described above for infected mice. A control group of two mice was treated with corn oil/2.5% DMSO only, and another two mice served as non-treated controls. Following euthanasia, the spleens were collected, and splenocytes were isolated and processed as described below.

### 2.6. Determination of the Parasite Load in Brain, Eyes and Heart Tissues by Real-Time PCR

The NucleoSpin DNA RapidLyze Kit (Macherey-Nagel, Oensingen, Switzerland) was used to isolate genomic DNA. DNA concentrations were determined using the QuantiFluor double-stranded DNA (dsDNA) system (Promega, Madison, WI, USA) [42]. After adjusting the DNA concentrations to 5 ng/μL, qPCR reactions were performed in a final volume of 10 μL containing 1x SensiFast master mix (Bioline, Meridian Bioscience, Greifensee, Switzerland), 0.5 μM of reverse and forward primers, 0.1 μM of 529rpeQ-P probe, 0.3 mM dUTP and one unit of heat-labile Uracil DNA Glycosylase (UDG) [42]. Fragments were amplified in the Light Cycler^®^ System (Roche, Basel, Switzerland) as previously mentioned [39,43]. Based on a standard curve consisting of a 10-fold serial dilution of DNA from *T. gondii* (tachyzoite numbers ranging from 1 × 10^4^ to 1 per 4 μL), the number of parasites was determined, and the parasite load was expressed as number of tachyzoites per 20 ng of DNA. Statistical analyses were performed using Microsoft Excel® software package (Microsoft, Redmond, WA, USA). Comparisons of the parasite burdens between groups were conducted with the Student *t*-test; *p*-values below 0.05 were considered statistically significant.

### 2.7. Assessment of Susceptibility of Murine Splenocytes to C3 and C4 In Vivo: Measurement of Viability and Proliferation of Splenocytes from Infected and Naîve Mice

These experiments were conducted as described by Boubaker et al. [39]. Briefly, spleens were aseptically collected under the hood after euthanasia, from mice treated with the compounds. Splenocytes were isolated from the spleens and seeded in 96-well plates (5 × 10^4^ cells/well). For proliferation/viability assays, splenocytes were either left unstimulated or were stimulated with Concanavalin A (ConA, 5 μg/mL), Lipopolysaccharide (LPS, 10 μg/mL), ConA plus C3 or C4 (0.1–2 μM) or LPS plus C3 or C4. The experiments were carried out in quadruplicate in 200 μL wells, and cultures were maintained at 37 °C/5% CO_2_ for 72 h. The proliferative response of splenocytes was measured using a BrdU cell proliferation kit (QIA58, Merck Millipore, Burlington, MA, USA) according to the instructions provided by the manufacturer, and absorbance measurements were obtained at 450/540 nm in a Hidex Sense multimode plate reader (Agilent Technologies, Santa Clara, CA, 515 USA).

To measure the impact on viability, an AlamarBlue assay was used [39]: resazurin (0.1 mg/mL) was added, and the fluorescence intensity was measured at 530/590 nm at 0, 1, 2, 3, 4 or 5 h. Differences were calculated by subtracting time point 0 values from each time point. Data are presented as mean of emission +/− SD for the indicated numbers. Data comparisons between groups were performed using a Student’s t-test.

## 3. Results

### 3.1. Treatments with C3 and C4 Do Not Affect the Viability of In Vitro Cultured Murine Splenocytes

**Figure 1:**
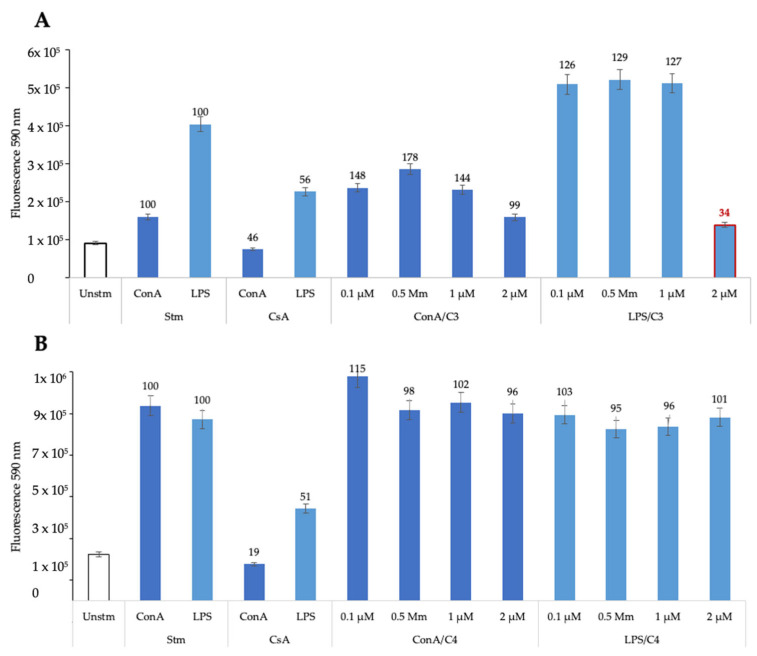
Effects of C3 (**A**) and C4 (**B**) on viability of murine T cells and B cells in vitro. Briefly, 96-well plates were seeded with splenocytes obtained from murine spleens (2 × 10^6^ cells/mL, 100 μL/well) and were left unstimulated (Unstm columns) or exposed to ConA (5 μg/mL) or LPS (10 μg/mL) (Stm columns). Splenocytes stimulated with ConA or LPS were also treated with cyclosporin A (CsA), a known inhibitor of B and T cell stimulation (third columns in the graphs). C3 and C4 were added at 0.1, 0.5, 1 and 2 μM, respectively, and cultivation was carried out for 48 h at 37 °C/5% CO_2_. Viability was assessed by resazurin reduction and is given as relative fluorescence units (RFUs).

We have shown previously that exposure of confluent HFF monolayers to C3 and C4 does not induce any viability impairment at concentrations up to 25 µM [38]. Here, (see Figure 1A), we observed that C3 reduced B cell viability only at 2 μM (highlighted in red), while C4 did not affect T or B cell viability at any concentration tested (Figure 1B). Based on these results, in vivo studies using a murine model of chronic toxoplasmosis were planned to evaluate C3 and C4 efficacy in a more complex biological environment, paving the way for future therapeutic development.

### 3.2. Effects of C3 and C4 on Early Zebrafish (Danio rerio) Embryo Development

The effects of C3 and C4 on early zebrafish embryo development were assessed in a blinded manner through daily microscopic inspection of 20 fertilized eggs per drug and concentration, cultured from 24 to 96 hpf in the presence or absence of 0.2 up to 20 µM of compounds. Results showed that both compounds induce embryotoxicity in a dose-dependent manner. C3 exhibited toxicity starting from 1 μM up to 20 µM, while C4 was toxic at all concentrations tested (Supplementary Table S1). Based on these results, in vivo studies using a murine model of congenital toxoplasmosis were excluded due to an anticipated increased risk for pregnancy interference.

### 3.3. Efficacy of C3 and C4 Treatment in CD1 Mice Infected with T. gondii Oocysts

**Figure 2:**
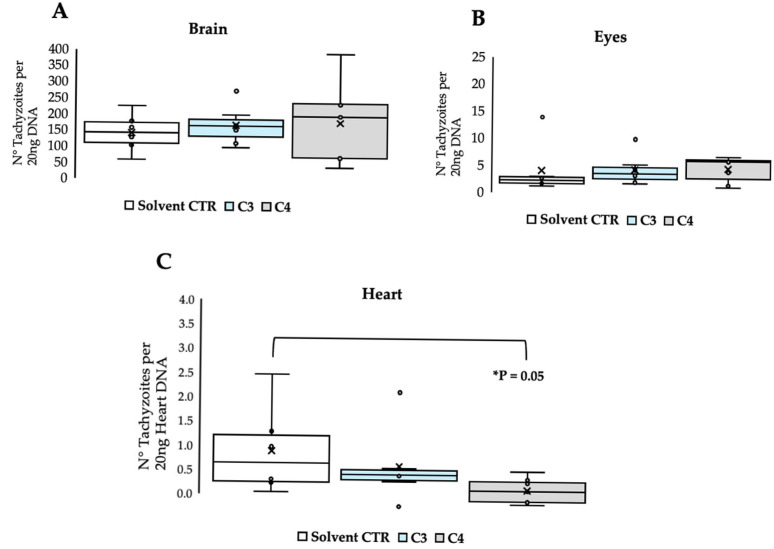
In vivo efficacy of C3 and C4 in CD1 mice experimentally infected with TgShSp1 oocysts. Box plots show the parasite load at 30 days p.i. in placebo control mice (solvent CTR, corn oil 2.5% DMSO) and C3- and C4-treated mice. The number of parasites in 20 ng DNA extracted from brain (**A**), eyes (**B**) and heart (**C**) was determined using qPCR. Parasite load data are represented as box-and-whisker (min to max) plots with the x showing the mean per group. Statistics were obtained using Student *t*-test, and *p*-values below 0.05 are considered statistically significant.

Outbred CD1 mice were experimentally infected by oral administration of 120 TgShSp1 type II oocysts and treated at 3 days p.i. with C3 and C4 for 5 days. Neither infection nor treatment caused clinical signs, indicating non-toxicity at this dosage. As shown in Figure 2A, C3 and C4 treatments did not reduce the chronic parasite load compared with that in the placebo control group (*p* = 0.45 and *p* = 0.54). Similarly, Figure 2B shows no reduction in the eye parasite load compared with that in the control group (*p* = 0.90 and *p* = 0.78.) However, the parasite load in the heart was significantly reduced in the C4-treated group (*p* = 0.02) (Figure 2C). Completed data are reported in Supplementary Table S2.

### 3.4. Assessment of Viability and Proliferative Capacity of Murine Splenocytes from T. gondii-Infected Mice Treated with C3 and C4 In Vivo

To assess whether C3 and C4 treatments affect B and T cell proliferation in these *T. gondii*-infected mice, splenocytes were isolated, cultured in vitro and stimulated with ConA or LPS to induce T and B cell proliferation, respectively. As shown in Supplementary Figure S1, C3 or C4 treatment did not affect either the viability or the proliferation of B and T cells of *T. gondii*-infected mice. Similar results were obtained using splenocytes from naîve mice treated with C3 and C4 (Supplementary Figure S1).

### 3.5. Assessment of Susceptibility of Murine Splenocytes to C3 and C4 In Vivo Treatments in Non-Infected Mice: Measurement of Viability and Proliferation

To investigate whether in vivo treatment with C3 and C4 could have had an impact on B or T cell viability or proliferation, non-infected mice were similarly treated either with C3, C4 or with vehicle alone, and isolated spleen cells were cultured in vitro and stimulated with either ConA or LPS. As seen in Supplementary Figure S2, C3 and C4 treatments in vivo had no effects on the viability and proliferative capacity of these splenocyte populations.

## 4. Discussion

In a previous study we reported that the TSC–gold complex (C3) and its TSC ligand (C4) selectively inhibited *T. gondii* growth in vitro at very low concentrations (nanomolar range) [38]. In this study the activities of C3 and C4 were assessed in a murine model of chronic toxoplasmosis. Prior to embarking on in vivo experiments, it was necessary to investigate the safety of these two compounds. While C3 and C4 did not affect the viability of human foreskin (HFF) monolayers in vitro at concentrations up to 25 µM [38], we wanted to know whether C3 or C4 could potentially impact on the viability of highly proliferative immune cells, which would then be detrimental to immunity. Splenocytes were thus isolated and cultured in vitro, and ConA and LPS were added to induce the proliferation of T and B cells, respectively. Treatments of proliferating B cells did not result in viability impairment, at any of the concentrations tested. The viability of T cells was reduced only at the highest concentration of C3 (2 µM). Thus overall, no pronounced immunosuppressive effects were noted at the highest drug concentrations. Another important safety issue is potential interference in embryo development. Congenital toxoplasmosis can occur upon a primary infection during pregnancy and potentially affects fetal health causing serious malformations or even abortion [44,45]. Therefore, any drug that affects embryo development can interfere in pregnancy outcome. We applied a zebrafish embryo development model that allows the determination of potential adverse effects imposed on embryos within the first 96 h postfertilization [41]. This model has shown considerable potential in terms of predicting drug-induced pregnancy interference in mice when using compound classes such as bumped kinase inhibitors (BKIs, drugs targeting apicomplexan calcium-dependent protein kinase 1) [41,46], and sulfadoxine–ruthenium complexes [39]. Thus, based on the detrimental effects on embryo development seen with C3 at 1 µM and C4 already at 0.2 µM, we did not consider these compounds suitable for use in pregnant mice. Efficacy in the murine model of chronic toxoplasmosis was assessed through the quantification of parasite burden in the brain, eyes and heart. Neither compound was effective in reducing the parasite burden in the brain and in the eyes. This lack of efficacy of C3 and C4 is likely due to the inability of these compounds, similarly to many other drugs tested for toxoplasmosis, to cross the blood–brain barrier (BBB). For instance, despite its significant tissue penetration, spiramycin shows poor ability to cross the BBB and fails to reach effective concentrations in the brain. This is due to the presence of efflux transporters such as multidrug-resistant protein 2 (Mrp2) and P-glycoprotein, which actively transport spiramycin, and other macrolide antibiotics, out of the brain [47,48]. Nevertheless, a significant decrease in the parasite burden was observed in the hearts of mice infected with *T. gondii* and treated with C4, even though the infection rate in this organ at the time of euthanasia was very low, including in the infected group treated with the solvent only. Indeed, it has been reported that the parasite burden in the hearts of mice is usually undetectable after approximately 2 weeks postinfection [49]. However, this insight might not be carved in stone. A recent report on the efficacy of a trithiolato–diRuthenium complex conjugated with sulfadoxine in *T. gondii*-oocyst-infected mice also indicated reduced parasite load in the eyes and in the heart tissues of treated mice [39]. While in the present study mice were euthanized at 30 days p.i., the timing of parasitic spread can be influenced by factors such as the route of administration of the parasites and the strain of *T. gondii* used for infection [50]. However, the significant reduction in parasite load observed in the hearts of mice treated with C4, the TSC ligand lacking the gold moiety, suggests that it has greater potential for further therapeutic development against toxoplasmosis than C3. Moreover, the compound does not affect the viability and proliferation of murine splenocytes following in vivo treatment. A previous study demonstrated that in vivo treatment with two (*E*)-2-(1-(4-chlorophenylthio) propan-2-ylidene)-hydrazine carbothioamides prolonged the survival of *T. gondii*-infected wild-type mice, and this was associated with increased IFN-γ and IL-12 production [37], highlighting the potential of this chemical class as promising therapeutic candidates for toxoplasmosis treatment. Since efficacy against parasite burden in the present study was observed only in the heart, further studies should also investigate additional organs and explore alternative routes of administration and formulations that could improve the compounds’ activity, together with evaluation of pharmacokinetics to better understand their metabolism and optimize their therapeutic potential. It would also be interesting to test TSCs in combination with standard drug treatments, such as sulfadiazine or pyrimethamine [51,52], and to investigate whether treatment efficacy can be improved. Indeed, combination protocols have been suggested, for example, with artemisone and artemiside, which were reported as being partially active in a murine model of reactivated toxoplasmosis. However, even though these two compounds controlled the initial phases of reactivated infection, mice died within 25 days after the treatment was discontinued, indicating that the parasite was not fully eradicated [53]. More recently, artemisone combined with a bumped kinase inhibitor (BKI-1748) showed synergistic effects against *T. gondii* in vitro. However, the synergistic effect could not be reproduced in CD1 mice experimentally infected with *T. gondii* oocysts [54]. On the other hand, a combined treatment with BKI-1748 and endochin-like quinolones of pregnant mice infected with the closely related apicomplexan *Neospora caninum* resulted in improved efficacy compared with treatments with individual drugs [55]. In any case, combination treatments can improve efficacy against the parasites and reduce toxicity compared with when the molecules were used alone. Detailed in vitro studies combined with affinity-based target identification have shown that C3 and C4 bind a large number of ribosomal proteins, suggesting that they both interfere in protein biosynthesis. However, long-term in vitro treatments revealed that tachyzoites readily adapted to high concentrations (>10-fold IC_50_ value) of these compounds, resulting in loss of efficacy and increased parasite tolerance towards treatments. However, this tolerance to these two compounds was rapidly lost when the drugs were removed, and the parasites reverted to their original drug sensitivity within a few days. Proteomic analyses of C3- and C4-adapted strains demonstrated that this transient adaptation phenomenon was associated with the upregulated expression of tachyzoite membrane proteins and transporters [38]. Whether this transient adaptation also occurs in vivo is not known, but if yes, it could also contribute to the lack of efficacy of these two TSCs. Finally, the results of the present study agree with those of several previous studies showing that the addition of metals does not always enhance the activity of TSCs. A study on murine and human tumor cells reported that the ligands and their Cu(II), Ni(II), Zn(II) and Cd(II) complexes exhibited similar activity in leukemia and lymphoma cell lines in suspension [56]. In contrast, a more recent study assessing ferrocenyl and ruthenocenyl derivatization of the benzimidazole drug albendazole showed that this resulted in a complete abolishment of activity against the protozoan parasite *Giardia lamblia* [57]. In contrast, against *T. gondii*, some ferrocene albendazole analogues exhibited better in vitro activity than albendazole and showed lower host cell toxicity. A classic example of a ferrocene drug for the treatment of apicomplexans is ferroquine, a derivative of the anti-malarial chloroquine, and ferroquine is now in phase II clinical studies for malaria treatment [57].

Currently, reliable and efficient in vitro drug screening systems are available for *T. gondii*; however, in vivo preclinical screening models still require improvement. As previously stated [39], pharmacokinetic and pharmacodynamic studies are essential to guide the design of in vivo experiments. Such studies will help address the question of why most drug candidates identified in vitro fail during the preclinical phase. In conclusion, this study underscores the importance of continued research into thiosemicarbazones and related compounds for toxoplasmosis treatment, highlighting their potential as therapeutic candidates despite the current limitations. It also emphasizes the need for further investigation into alternative administration routes, combination therapies and pharmacokinetics to overcome challenges such as the blood–brain barrier.

## Data Availability

Data are contained within the article and Supplementary Materials.

## Supplementary Materials

The following supporting information can be downloaded at https://www.mdpi.com/article/10.3390/biomedicines13081879/s1, Figure S1: Effects of C3 and C4 plus infection on viability of murine T-cells and B-cells in vivo; Figure S2: Effects of C3 and C4 on viability of murine T-cells and B-cells in vivo; Table S1: Effects of C3 and C4 on Early Zebrafish (Danio rerio) Embryo Development; Table S2: Efficacy of C3 and C4 Treatment in CD1 Mice Infected with T. gondii Oocysts (qPCR results).
