# Supplementary material for: In Vivo Safety and Efficacy of Thiosemicarbazones in Experimental Mice Infected with Toxoplasma gondii Oocysts
Source: Biomedicines. 2025 Aug 1;13(8):1879. doi: 10.3390/biomedicines13081879 (PMC12383829; doi:10.3390/biomedicines13081879)
Supplement: Supplementary file 1 [file biomedicines-13-01879-s001.zip › Supplementary Figures.pdf]

Supplementary Figures

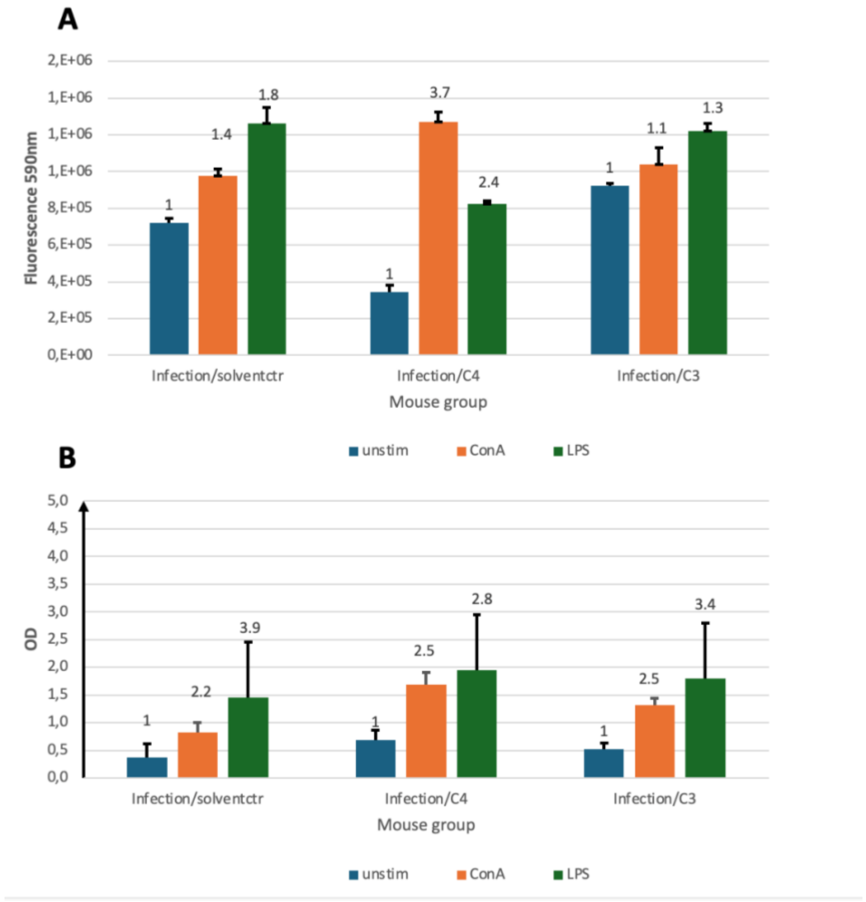

Suppl. Figure S1 (A) Effects of C3 and C4 plus infection on viability of murine T-cells and B-cells *in vivo.* 96-well plates were seeded with splenocytes obtained from murine spleens (2× 10^6 cells/mL, 100 µL/well) of mice infected and treated with C3 and C4, respectively, and were exposed to ConA (5 µg/mL) or LPS (10 µg/mL) *in vitro*. Cultivation was carried out for 48 h at 37 °C/5% CO_2. Viability was assessed by resazurin reduction and is given as relative fluorescence units (RFU). (B) Effects of C3 and C4 plus infection on proliferation of murine T-cells and B-cells *in vivo*. Stimulation was done as explained above and proliferation was measured using BrdU-ELISA.

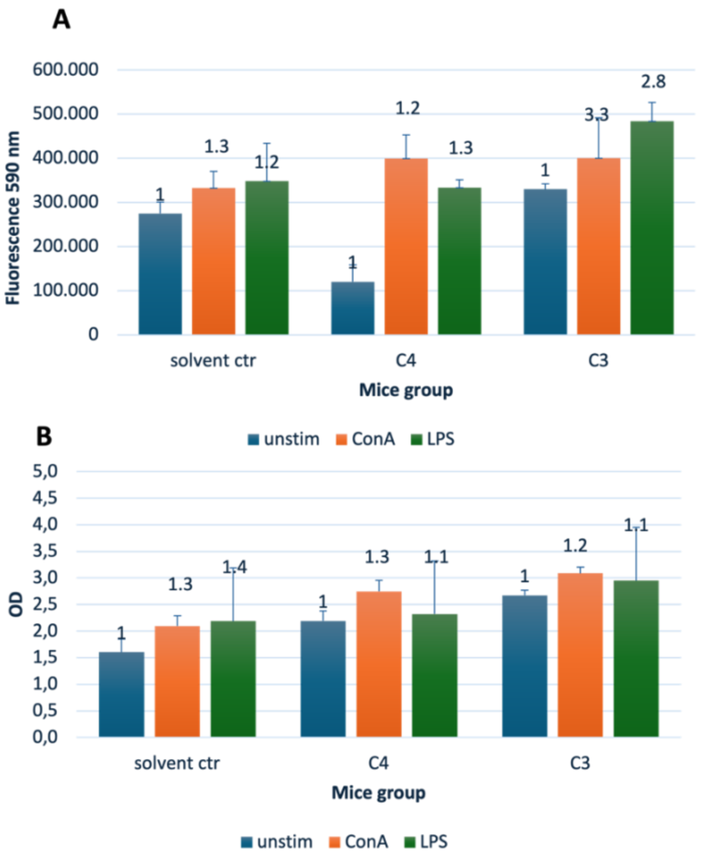

Suppl. Figure S2. (A) Effects of C3 and C4 on viability of murine T-cells and B-cells *in vivo.* 96-well plates were seeded with splenocytes obtained from murine spleens (2× 10^6 cells/mL, 100 µL/well), of mice treated with C3 and C4, respectively and were exposed to ConA (5 µg/mL) or LPS (10 µg/mL) *in vitro*. Cultivation was carried out for 48 h at 37 ° C/5% CO_2. Viability was assessed by resazurin reduction and is given as relative fluorescence units (RFU). (B) Effects of C3 and C4 on proliferation of murine T-cells and B-cells *in vivo*. Stimulation was done as explained above and proliferation was measured using BrDU-ELISA.
